# Sedentary behaviour among general practitioners: a systematic review

**DOI:** 10.1186/s12875-020-01359-8

**Published:** 2021-01-04

**Authors:** Richard S. Mayne, Nigel D. Hart, Neil Heron

**Affiliations:** 1 Centre for Medical Education, School of Medicine, Dentistry and Biomedical Sciences, Queen’s University Belfast, Belfast, UK; 2 Centre for Public Health, School of Medicine, Dentistry and Biomedical Sciences, Queen’s University Belfast, Belfast, UK

**Keywords:** General practice, Primary care, Sedentary behaviour, Systematic review, Physical activity

## Abstract

**Background:**

Sedentary behaviour is when someone is awake, in a sitting, lying or reclining posture and is an independent risk factor for multiple causes of morbidity and mortality. A dose-response relationship has been demonstrated, whereby increasing sedentary time corresponds with increasing mortality rate. This study aimed to identify current levels of sedentary behaviour among General Practitioners (GPs), by examining and synthesising how sedentary behaviour has been measured in the primary care literature.

**Methods:**

A systematic review was conducted to identify studies relating to levels of sedentary behaviour among GPs. Searches were performed using Medline®, Embase®, PscycINFO, Web of Science and the Cochrane Library, from inception of databases until January 2020, with a subsequent search of grey literature. Articles were assessed for quality and bias, with extraction of relevant data.

**Results:**

The search criteria returned 1707 studies. Thirty four full texts were reviewed and 2 studies included in the final review. Both were cross-sectional surveys using self-reported estimation of sedentary time within the International Physical Activity Questionnaire (IPAQ). Keohane et al. examined GP trainees and GP trainers in Ireland. 60% reported spending in excess of 7 h sitting each day, 24% between 4 and 7 h, and 16% less than or equal to 4 h. Suija et al. examined female GPs in Estonia. The mean reported daily sitting time was 6 h and 36 min, with 56% sitting for over 6 h per day. Both studies were of satisfactory methodological quality but had a high risk of bias.

**Conclusion:**

There is a paucity of research examining current levels of sedentary behaviour among GPs. Objective data is needed to determine GPs’ current levels of sedentary behaviour, particularly in light of the increase in remote consulting as a result of the COVID-19 pandemic.

**Supplementary Information:**

The online version contains supplementary material available at 10.1186/s12875-020-01359-8.

## Background

**Fig. 1:**
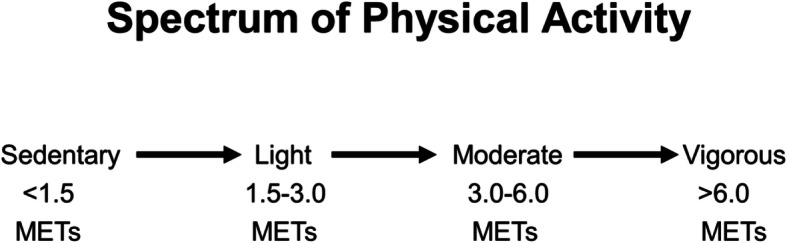
Spectrum of physical activity

Sedentary behaviour is when someone is awake, in a sitting, lying or reclining posture, in a state of low energy expenditure, typically expending less than 1.5 metabolic equivalent of tasks (METs) [1, 2]. METs allow comparisons to be made between the energy expended during different states [3]. METs are calculated as a ratio of the rate of energy expended during an activity compared to the rate of energy expended at rest [3]. For example, 1.0 METs is the rate of energy expenditure while sitting at rest [3]. A 2.0 METs activity, such as ironing, expends twice the energy used by the body when sitting at rest [3]. Physical activity is any movement of the body produced by skeletal muscles that requires energy expenditure [4]. Physical activity can therefore be viewed as a spectrum, ranging from sedentary behaviour to light, moderate and vigorous physical activity (Fig. 1.). Physical inactivity is a separate entity, instead defined as when an individual has insufficient levels of physical activity, i.e. less than current physical activity recommendations [2, 5].

The effect of sedentary behaviour on health has been an area of interest among researchers since the pioneering work of the epidemiologist, Jeremy Morris, in the 1940s and 1950s. Morris and colleagues demonstrated that sedentary bus drivers had higher rates of mortality due to coronary heart disease than bus conductors, their more active colleagues [6, 7]. Since then, there has been an ever-increasing weight of evidence to demonstrate the negative health effects of sedentary behaviour [8]. It is now acknowledged that sedentary behaviour is associated with multiple adverse health outcomes, including mental health issues, obesity, type 2 diabetes, multiple forms of cardiovascular disease and dementia, as well as breast, colorectal, endometrial and ovarian cancer [8–12]. As a result of these adverse health outcomes, sedentary behaviour is associated with increased all-cause mortality, even when allowing for confounding variables [12–15]. These findings demonstrate a dose-response relationship, whereby increasing sedentary time corresponds with increasing mortality rate [12–15]. Sedentary behaviour has significant economic costs. Sedentary behaviour was estimated to cost the United Kingdom (UK) National Health Service (NHS) £0.7 billion in 2016–2017 [16]. A total of 69,276 deaths could potentially have been avoided in the UK if sedentary behaviour was eliminated [16]. In light of these findings, 2019 UK physical activity guidelines state that through all stages of life, individuals should minimise their sedentary behaviour, and break up periods of sedentary behaviour where possible [5].

Previous studies have examined levels of sedentary behaviour among other professions [17, 18], however General Practice is a different working environment, with different challenges and opportunities from other professions, even within the field of healthcare. Primary care has been described as “the cornerstone” of the NHS, providing over 300 million patient consultations per year [19, 20]. This enables General Practitioners (GPs) to play an important role in both primary and secondary prevention, by providing evidence-based lifestyle guidance to patients. GPs can reinforce important public health messages among their patients, making them more specific, individualised and personally relevant. Numerous studies have demonstrated that GPs who are more physically active are more likely to recommend physical activity to their patients [21–30]. Patients are also more likely to make healthy lifestyle changes recommended by their doctor if they believe their doctor follows the health advice themselves [31–34]. It could therefore be argued that reducing sedentary behaviour and increasing physical activity among GPs could lead to health benefits for both GPs themselves, at an individual level, and their patients, at a population level. Within the context of day-to-day General Practice, this would primarily involve interrupting or replacing prolonged periods of sitting with physical activity. One example is the use of active workstations, such as standing desks, combined with short breaks for physical activity, such as “exercise snacks”. Sitting while using a computer or telephone is a form of sedentary behaviour (≤1.5 METs), whereas standing while using a computer or telephone is a form of light physical activity (1.8 METs) [35]. Reducing sedentary behaviour among GPs, by replacing sedentary behaviour with physical activity, could therefore play a vital role, as part of a multifaceted approach alongside public health initiatives and changes to the built environment, in ensuring a culture shift away from an increasingly sedentary society, towards an increasingly physically active society.

The aim of this systematic review is to identify the current levels of sedentary behaviour among GPs. The review examines and synthesises how sedentary behaviour has been measured in the primary care literature.

## Methods

This systematic review was conducted according to Preferred Reporting Items for Systematic Reviews and Meta-Analyses (PRISMA) guidance. The focus of this review was the identification of peer-reviewed, published articles which reported sedentary behaviour among GPs (including family doctors and primary care doctors and/or physicians). Searches were performed using Medline®, Embase®, PscycINFO and Web of Science databases, with assistance from a medical librarian (last search performed on 29th January 2020). Given the low number of eligible studies identified, a subsequent search of the Cochrane Library database, as well grey literature within thesis, dissertation and clinic trial databases (OpenGrey, EThOS, DART-Europe, OATD, International Clinical Trials Registry Platform) was performed, with hand-searching of reference lists of screened studies. Terms relating to General Practice and sedentary behaviour were combined using keywords, title, or abstract, with appropriate alternative spellings and truncation symbols. Due to the small number of available studies identified, a narrative synthesis was undertaken of the included studies.

### Study selection

Detailed searches were performed within Medline®, Embase®, PscycINFO, Web of Science and Cochrane Library databases, as well grey literature within thesis, dissertation and clinic trial databases (OpenGrey, EThOS, DART-Europe, OATD, International Clinical Trials Registry Platform), supplemented by hand-searching of reference lists of screened studies. Two authors independently screened titles and abstracts of publications retrieved from the completed searches, once duplicates were removed. A third author was available to resolve any conflicts in study inclusion. Articles were discarded if they did not meet the inclusion criteria, with a record kept of the number discarded at each stage and reason for exclusion. Although no language restrictions were made, all included papers were written in English. Extracted data included populations and settings, sample sizes and response rates, methodological issues, eligibility criteria, study design, and definitions and measures. The terms ‘general practitioner’, ‘GP’, ‘family physician’, and ‘family practitioner’ were all considered to relate to the same discipline. For the purposes of this study, the term used is ‘general practitioner’ or ‘GP’.

### Data synthesis and quality assessment

Data were synthesised in terms of reported hours of sedentary behaviour among study participants. Objective criteria were used to assess quality and risk of bias within recruitment, sample population, reliability and validity of outcome measures according to the Newcastle-Ottawa quality assessment scale adapted for cross sectional studies, as previously described by Herzog et al. [36] and Luchini et al. [37] (additional file 1).

## Results

**Fig. 2:**
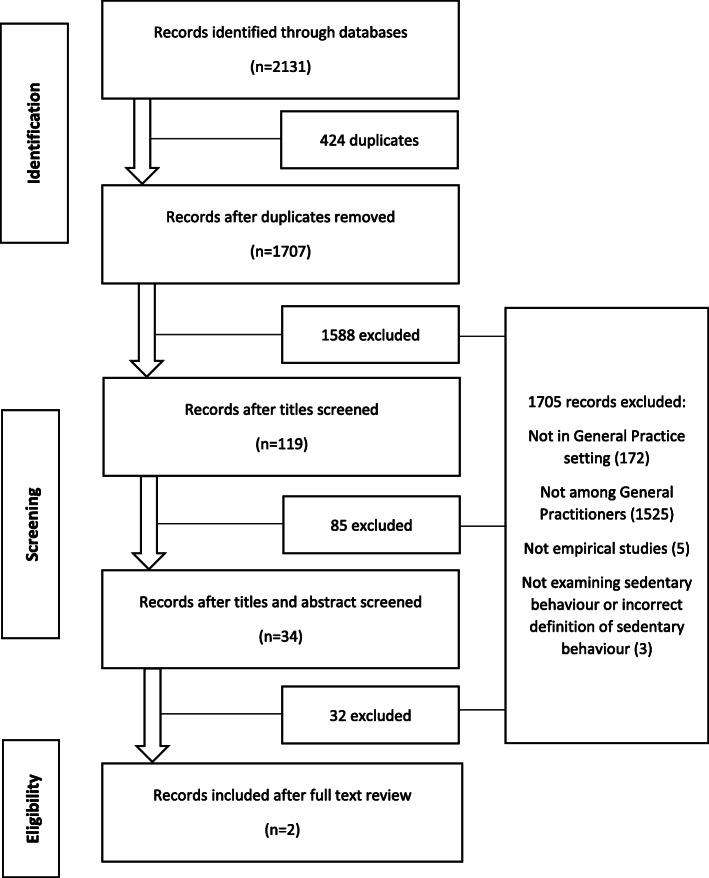
Flow diagram for identification, screening, eligibility, and inclusion of papers for review

One thousand seven hundred and seven studies were identified after duplicates were removed. After screening titles and abstracts, 1673 were excluded. Out of 34 full text articles which were reviewed, only 2 measured sedentary behaviour among GPs, both of which were included in the final review (Fig. 2). Applying the Newcastle-Ottawa quality assessment scale adapted for cross sectional studies, both included studies were of satisfactory methodological quality (Table 1). The main reasons for study exclusion were studies not taking place in the General Practice setting, studies examining patients, not GPs themselves, and studies not examining sedentary behaviour. Although 5 studies initially appeared to relate to levels of sedentary behaviour among GPs, 3 of these were excluded as they used an incorrect, imprecise or outdated definition of sedentary behaviour [22, 23, 40]. Brotons et al. [22], Cornuz et al. [23] and Jonsdottir et al. [40] did not clearly state how they defined GPs as being sedentary. It appears that they were instead referring to GPs who did not exercise regularly, who would currently be defined as being physically inactive (ie. not meeting physical activity recommendations). A description of the studies included in the final review is displayed in Table 2.

**Table 1:** Quality assessment of the included studies based on the Newcastle-Ottawa quality assessment scale adapted for cross sectional studies

Study	Design	Selection (max. 5*)				Comparability (max. 2*)	Outcome (max. 3*)		Total score (max. 10*)
		Representativeness of the sample	Sample size	Nonrespondents	Ascertainment of exposure	Based on design and analysis	Assessment of outcome	Statistical test	
Keohane et al. [38]	Cross-sectional	*	*		**	*	*		6*
Suija et al. [39]	Cross-sectional	*	*		**	*	*		6*
**Scoring** • Very Good Studies: 9–10 stars • Good Studies: 7–8 stars • Satisfactory Studies: 5–6 stars • Unsatisfactory Studies: 0 to 4 stars

**Table 2:** Description of Included Studies

Author	Country	Number of participants	Study design	Criteria for inclusion	Assessment method	Objectivity	Quality
Keohane et al.	Ireland	219	Cross-sectional	GP Trainers and GP Trainees	Self-reported questionnaire	Non-objective	Satisfactory
Suija et al.	Estonia	198	Cross-sectional	Female GPs	Self-reported questionnaire	Non-objective	Satisfactory

The first study included was a cross-sectional survey of all GP trainees and GP trainers in the Republic of Ireland by Keohane et al. [41]. In total there were 219 eligible respondents [41]. The primary aim of the study was to explore levels of physical activity among Irish GPs and GP trainees, with an additional aim of investigating their perceived barriers to exercise [41]. The study used the self-administered International Physical Activity Questionnaire (IPAQ) to assess levels of physical activity [41–43]. Within the short form of the IPAQ, although it is primarily a tool used for self-estimation of physical activity, there is a question relating to sedentary behaviour [42]. Participants are asked about the time they spend sitting on a weekday while at work, at home, while doing course work and during leisure time, which may include time spent sitting at a desk, visiting friends, reading or sitting or lying down to watch television [42]. In the study by Keohane et al., 60% reported spending in excess of 7 h sitting each day, 24% between 4 and 7 h, and 16% less than or equal to 4 h [41]. There was no significant difference in sitting time between male and female respondents (*p*=0.61) [41]. There was, however, a statistically significant difference in sitting time reported by trainees working in hospital compared to those working in GP Practices (*p*< 0.05) and between qualified GPs and GP trainees (*p*< 0.05) [41]. There was no specific detail of the mean levels of sitting time within each of these groups [41]. It is likely that trainees working in the hospital setting were overall less sedentary than those working in the GP Practice setting, and therefore GP trainees were overall less sedentary than qualified GPs, however, in the absence of sufficient data we cannot say this with certainty [41].

The second study included was a cross-sectional survey of female GPs in Estonia [44]. There were 198 responses included in the analysis [44]. The aim of this study was to explore physical activity among Estonian GPs, as well as their physical activity counselling practices [44]. Only female GPs were included, as 95% of GPs in Estonia were female at the time of the study [44]. The self-administered International Physical Activity Questionnaire (IPAQ) short form was translated into Estonian and used to assess self-reported levels of physical activity, as well as sitting time [42–44]. The mean amount of daily sitting time was 6 h and 36 min, with 56% sitting for over 6 h per day [44]. Levels of physical activity were compared between those who reported sitting less than 6 h per day and those who reported sitting more than 6 h per day [44]. Although those who reported sitting less than 6 h per day appeared to be slightly more physically active, this was not statistically significant (*p*=0.207) [44].

## Discussion

### Overview

This is the first systematic review of the levels of sedentary behaviour among GPs. One thousand seven hundred and seven studies were identified from our search criteria, with 2 studies included in the final review. Included studies were cross-sectional, with self-reporting of sedentary behaviour in hours and minutes. Both studies were of satisfactory methodological quality, however both had risk of bias and lack of objectivity. They both focused primarily on levels of physical activity among GPs, using the International Physical Activity Questionnaire (IPAQ). In the IPAQ, just one question concerns sedentary behaviour, where participants are asked to estimate how much time they spent sitting on a week day [42]. Both studies may have been affected by selection bias, whereby survey participants may have been less sedentary and more physically active than those who did not respond. The study by Suija et al. questioned only female GPs, so findings may not be valid among male GPs, however there were no significant differences between males and females in the study by Keohane et al. [44]. As responses were self-estimated, as oppose to objectively measured findings, participants may also have either overestimated or underestimated their true levels of physical activity and sedentary behaviour. Both studies used validated questionnaires for the self-assessment of physical activity and sedentary behaviour. There is some debate regarding the validity of self-estimated, compared to objectively measured, findings of sedentary behaviour and physical activity [38, 39, 42, 43, 45–49]. It is widely acknowledged that objective data (such as that obtained using accelerometers or pedometers) has higher validity than self-estimation of sedentary behaviour and physical activity, with self-estimation shown to typically underestimate sedentary behaviour by approximately 1.74 h per day [45, 46, 50]. With the recent increase in remote consulting among GPs as a result of the COVID-19 pandemic, GPs have less face-to-face interaction with patients, with the majority of consultations now happening via telephone and video [51]. This opens up both challenges and opportunities for GPs regarding their levels of sedentary behaviour [52, 53]. It does, however, mean that GPs now have more in common with workers in other medical and non-medical environments, such as office and call centre workers, where interventions targeted at reducing levels of sedentary behaviour have had varying levels of success [54–58].

### Strengths and limitations

Strengths of this systematic review were the use of a clearly defined search and study selection strategy, with double reviewing of all stages. Using a wide search strategy, with no exclusion based on language, supplemented by hand-searching of reference lists, allowed authors to identify as many eligible studies as possible. Despite this, just 2 eligible studies were identified, both in English. A limitation of this review is the lack of studies available in the area of sedentary behaviour among GPs. Sedentary behaviour is a novel and emerging area of research. Although there has been an increasing volume of research examining sedentary behaviour in other settings, this study has identified a lack of research in the field of General Practice. Most studies in the General Practice setting appear to focus on either physical activity or sedentary behaviour of patients, not among GPs themselves.

### Conclusion

In light of the established associations between sedentary behaviour, adverse health outcomes and mortality, GPs should consider their own levels of sedentary behaviour, as well as that of their patients. GPs can potentially be key protagonists in reducing sedentary behaviour among the general population by virtue of their position in the healthcare system, where they have significant levels of patient contact and opportunities for health promotion.

At present, there is a paucity of research examining current levels of sedentary behaviour among GPs. This systematic review identified just 2 papers assessing levels of sedentary behaviour among GPs, both of which used self-reported estimations [41, 44]. Given that GPs who are more physically active are more likely to recommend physical activity to their patients, and patients are more likely to make healthy lifestyle changes if they believe their doctor follows the health advice themselves, by reducing their sedentary behaviour and increasing their physical activity, GPs could play an important role in the development of a less sedentary and more physically active society [31–34]. There is therefore a clear need for more reliable and objective data to determine the current levels of sedentary behaviour among GPs, particularly in light of the increase in remote consulting as a result of the COVID-19 pandemic.

## Additional Files

**Additional file 1.** Critical appraisal tool for cross-sectional studies. Modified from the Newcastle-Ottawa Quality Assessment Scale for Cohort Studies.

## Data Availability

The datasets used and analysed during the current study are available from the corresponding author following reasonable request.

## Abbreviations

COVID-19: Coronavirus disease 2019
GP: General Practitioner
IPAQ: International Physical Activity Questionnaire
METs: Metabolic Equivalent of Tasks
NHS: National Health Service
PRISMA: Preferred Reporting Items for Systematic Reviews and Meta-Analyses
