# Supplementary material for: Sedentary behaviour among general practitioners: a systematic review
Source: BMC Fam Pract. 2021 Jan 4;22:6. doi: 10.1186/s12875-020-01359-8 (PMC7779649; doi:10.1186/s12875-020-01359-8)
Supplement: Supplementary file 1 — Additional file 1. Critical appraisal tool for cross-sectional studies. Modified from the Newcastle-Ottawa Quality Assessment Scale for Cohort Studies. [file 12875_2020_1359_MOESM1_ESM.docx]

**Newcastle-Ottawa Quality Assessment Scale (adapted for cross-sectional studies)**

**Selection: (Maximum 5 stars)**

*1) Representativeness of the sample:*

a) Truly representative of the average in the target population. * (all subjects or

random sampling)

b) Somewhat representative of the average in the target population. * (nonrandom sampling)

c) Selected group of users.

d) No description of the sampling strategy.

*2) Sample size:*

a) Justified and satisfactory. *

b) Not justified.

*3) Non-respondents:*

a) Comparability between respondents and non-respondents characteristics is

established, and the response rate is satisfactory. *

b) The response rate is unsatisfactory, or the comparability between respondents

and non-respondents is unsatisfactory.

c) No description of the response rate or the characteristics of the responders and

the non-responders.

*4) Ascertainment of the exposure (risk factor):*

a) Validated measurement tool. **

b) Non-validated measurement tool, but the tool is available or described. *

c) No description of the measurement tool.

**Comparability: (Maximum 2 stars)**

1) *The subjects in different outcome groups are comparable, based on the study design or analysis. Confounding factors are controlled.*

a) The study controls for the most important factor (select one). *

b) The study controls for any additional factor. *

**Outcome: (Maximum 3 stars)**

*1) Assessment of the outcome:*

a) Accelerometery-measured objective data. **

b) Self report. *

c) No description.

*2) Statistical test:*

a) The statistical test used to analyze the data is clearly described and appropriate, and the measurement of the association is presented, including confidence intervals and the probability level (p value). *

b) The statistical test is not appropriate, not described or incomplete.

**Scoring:**

Very Good Studies: 9-10 points

Good Studies: 7-8 points

Satisfactory Studies: 5-6 points

Unsatisfactory Studies: 0 to 4 points

This scale has been adapted from the Newcastle-Ottawa Quality Assessment Scale for cohort studies to perform a quality assessment of cross-sectional studies for the systematic review, “Sedentary Behaviour Among General Practitioners: A Systematic Review.”
